# Assessing the requirements of prenatal UBE3A expression for rescue of behavioral phenotypes in a mouse model for Angelman syndrome

**DOI:** 10.1186/s13229-020-00376-9

**Published:** 2020-09-18

**Authors:** Monica Sonzogni, Peipei Zhai, Edwin J. Mientjes, Geeske M. van Woerden, Ype Elgersma

**Affiliations:** 1 Department of Neuroscience and the ENCORE Expertise Center for Neurodevelopmental Disorders, Erasmus MC University Medical Center, 3015 CN Rotterdam, The Netherlands; 2 Department of Neurology, The First Affiliated Hospital of Henan University, No.357, Ximendajie Street, Kaifeng City, Henan Province China

**Keywords:** Angelman syndrome, UBE3A, Mouse model, Behavior, Critical period, ASO therapy

## Abstract

**Background:**

Angelman syndrome (AS) is a rare neurodevelopmental disorder caused by the loss of functional ubiquitin protein ligase E3A (UBE3A). In neurons, UBE3A expression is tightly regulated by a mechanism of imprinting which suppresses the expression of the paternal *UBE3A* allele. Promising treatment strategies for AS are directed at activating paternal *UBE3A* gene expression. However, for such strategies to be successful, it is important to know when such a treatment should start, and how much UBE3A expression is needed for normal embryonic brain development.

**Methods:**

Using a conditional mouse model of AS, we further delineated the critical period for UBE3A expression during early brain development. *Ube3a* gene expression was induced around the second week of gestation and mouse phenotypes were assessed using a behavioral test battery. To investigate the requirements of embryonic UBE3A expression, we made use of mice in which the paternal *Ube3a* allele was deleted.

**Results:**

We observed a full behavioral rescue of the AS mouse model phenotypes when *Ube3a* gene reactivation was induced around the start of the last week of mouse embryonic development. We found that full silencing of the paternal *Ube3a* allele was not completed till the first week after birth but that deletion of the paternal *Ube3a* allele had no significant effect on the assessed phenotypes.

**Limitations:**

Direct translation to human is limited, as we do not precisely know how human and mouse brain development aligns over gestational time. Moreover, many of the assessed phenotypes have limited translational value, as the underlying brain regions involved in these tasks are largely unknown.

**Conclusions:**

Our findings provide further important insights in the requirement of UBE3A expression during brain development. We found that loss of up to 50% of UBE3A protein during prenatal mouse brain development does not significantly impact the assessed mouse behavioral phenotypes. Together with previous findings, our results indicate that the most critical function for mouse UBE3A lies in the early postnatal period between birth and P21.

## Background

Angelman syndrome (AS) is a neurodevelopmental disorder characterized by distinct features such as severe intellectual disability, absence of speech, jerky movements, hyperactivity, seizures, and EEG abnormalities [1]. The genetic cause underlying AS became apparent when a deletion of chromosome 15q11-q13 was identified on the maternally inherited allele [2, 3]. In contrast, the deletion of the paternal 15q11-q13 locus causes Prader-Willi syndrome (PWS), a disorder characterized by obesity and hyperphagia. The discovery of neuronal imprinting of chromosome 15q11-q13 explained the occurrence of two distinct disorders associated with the same deletion [2]. Kishino and colleagues later identified the ubiquitin protein ligase E3A (UBE3A) gene as the causal gene for AS [4].

Imprinting of UBE3A is regulated by the expression of an antisense RNA (*UBE3A-ATS*), which is expressed from the paternally inherited chromosome in the brain [5, 6]. No effective therapy is currently available for AS, but the discovery of UBE3A-ATS-dependent silencing of paternal *UBE3A* opened the door to pursue a treatment that involves the reactivation of the paternal gene [7, 8]. Clinical trials that use antisense-oligonucleotides (AONs/ASOs) to activate paternal *UBE3A* expression in individuals with AS have recently been initiated and offer a promising approach for treating AS.

For the clinical success of a paternal *UBE3A* gene reactivation approach, it is essential to know if UBE3A is critical for early brain development. If so, such a therapy might have to be given at or even before birth [9]. To address this question, we previously generated an inducible *Ube3a* mouse line which contains a transcriptional “STOP-cassette” flanked by LoxP sites (*Ube3a*^*tm1Yelg*^; from hereon called *Ube3a*^*LSL*^) allowing UBE3A expression after Cre-mediated deletion of the STOP cassette [10]. By restoring UBE3A expression at different time points, we observed that an early therapeutic intervention is needed to rescue the majority of AS phenotypes [10]. Specifically, we found that inducing *UBE3A* gene expression around the first embryonic day of development results in a full rescue of all phenotypes, whereas inducing *UBE3A* gene expression at P21 only rescued motor coordination. Neonatal gene reactivation resulted in a limited behavioral rescue, but these results should be interpreted with care, as we did not manage to get full gene reinstatement in newborn mice. Hence, the precise critical window for obtaining a full behavioral rescue in a mouse model of AS remains undetermined.

It is important to realize that both *Ube3a* alleles are expressed during early (prenatal) brain development as paternal *Ube3a* gene silencing is not complete until the first few days *afte*r birth [11]. If prenatal brain development requires both copies of *Ube3a* to be active, *Ube3a* gene reactivation after birth would have limited success. To specifically address the importance of bi-allelic *UBE3A* expression before birth, one can use mice in which the paternal allele is mutated, whereas the maternal gene is normally expressed. Mice lacking a functional copy of maternal *Ube3a* (m^−^/p^+^; “AS” mice) exhibit a number of robust phenotypes, including phenotypes that are directly relevant to AS [10, 12–16], but very few studies have investigated the effect of deleting the paternal *Ube3a* allele. Paternal loss of *Ube3a* expression affects the development of the cerebellum [12], but this does not have an impact on the motor performance [12, 13, 17, 18]. These findings are in line with a study that showed a limited role of the cerebellum in AS mice on these tasks [19]. However, it has been reported that deletion of both *Ube3a* alleles in mice affects licking behavior in a different way compared to maternal *Ube3a* deletion [17] which could suggest specific changes in cerebellar function.

The aim of this study is to further delineate the requirement of UBE3A expression during prenatal brain development. To address this, we used two approaches. First, we investigated a conditional mouse model of AS in which maternal *Ube3a* expression is reactivated before the last week of mouse embryonic development. Second, we investigated the importance of paternal *Ube3a* expression during prenatal brain development.

## Methods

### Mouse breeding

We made use of *Ube3a*^LSL^ (*Ube3a*^*tm1Yelg*^; MGI:5704099) mice as previously described [10]. These mice were maintained in the 129S2 background (full name: 129S2/SvPasCrl) by crossing male 129S2 *Ube3a*^*m+/p*LSL^ mice with 129S2 females. For the behavioral experiments, we used mice in the B6129S2F1 background, which were generated as described below. To generate embryonic reactivation of *Ube3a* at E12.5*,* 129S2 *Ube3a*^*m+/p*LSL^ female mice were crossed with Nestin-Cre-expressing male mice (Tg(Nes-cre)1Kln; MGI:2176173) [20] in the C57BL/6J background (Charles River Laboratories). This breeding yielded 4 experimental groups in a B6129SF1 background: *WT* mice with and without *Cre*, and *Ube3a*^*mLSL/p+*^ mice with and without *Cre.*

To test the role of paternal *Ube3a* on behavioral outcome measures, we made use of *Ube3a*^*tm1Alb*^ mice (MGI:2181811 )[12] (Fig. 3a) and *Ube3a*^*E113X*^ mutants (*Ube3a*^*tm2Yelg*^; MGI:5911277) [21] (Fig. 3b). *Ube3a*^*tm1Alb*^ mice were maintained (> 40 generations) in the 129S2 background by crossing male *Ube3a*^*m+/p−*^ mice with female 129S2 wild-type mice. *Ube3a*^*tm2Yelg*^ mice were maintained (> 20 generations) in the C57BL/6J (Charles River) background by crossing male *Ube3a*^*m+/pE113X*^ mice with female C57BL/6J wild-type mice. To generate mice lacking *Ube3a* on either the maternal or paternal or both alleles, we crossed female *Ube3a*^*tm1Alb*^ mice with male *Ube3a*^*tm2Yelg*^ mice. This breeding yielded 4 experimental groups: *WT* mice, heterozygous *Ube3a*^*m+/p−*^ mice, *Ube3a*^*m−/p+*^ (AS) mice, and homozygous *Ube3a*^*m−/p−*^ mice.

To test the contribution of both *Ube3a* alleles on UBE3A expression, we made use of *Ube3a*^*tm2Alb*^ mice in which UBE3A is fused to YFP (MGI:3771814) [22]. *Ube3a*^*tm2Alb*^ mice were maintained for > 20 generations in C57BL/6J, by crossing male *Ube3a*^*m+/pUbe3a-YFP*^ mice with female C57BL/6J wild-type mice. To generate mice expressing *Ube3a-YFP* on either the maternal or paternal allele, we crossed female *Ube3a*^*m+/pUbe3a-YFP*^ mice with male 129S2 wild-type mice, or male *Ube3a*^*m+/pUbe3a-YFP*^ with female 129S2 wild-type mice.

### Mouse husbandry

All mice were group-housed in a barrier facility, in individually ventilated cages (IVC; 1145 T cages from Techniplast). Mice were genotyped when they were 4–7 days old, and re-genotyped at the completion of the experiments. All animals were kept at 22 ± 2^0^C with a 12-h dark and light cycle, and provided with mouse chow (801727CRM(P) from Special Dietary Service) and water ad libitum*.* During behavioral testing, mice remained group-housed, except during the nest building test and subsequent forced swim test. Since we previously published a small but significant effect of sex on the rotarod and nest building test in a meta-analysis [14], we tried to balance the groups as much as possible (See Additional File, Table 1).

### Behavioral test battery

All behavioral experiments were performed during the light period of the light/dark cycle. Both male and female mice were used between 8 and 12 weeks of age. Mice were acclimatized to the testing room for 30 min before each behavioral performance. All behavioral testing and scoring were performed by an experimenter blind to genotype. Behavioral tests were precisely performed as previously described [14] and as listed below:

*Accelerating rotarod*. Motor capabilities were tested by placing the mice on the accelerating rotarod (4–40 rpm, in 5 min; model 7650, Ugo Basile Biological Research Apparatus, Varese, Italy). Mice were tested twice a day with a 45–60-min inter-trial interval for 5 consecutive days (same hour every day). For each day, the average time spent on the rotarod was calculated, or the time until the mouse made 3 consecutive wrappings/passive rotations on the rotarod (latency in seconds). Maximum duration of a trial was 5 min.

*Reversed rotarod.* Motor capabilities in the *Ube3a* paternal deficient line were tested by placing the mice on the accelerating rotarod (4–40 rpm, in 5 min; modified model 7650, Ugo Basile Biological Research Apparatus, Varese, Italy) in such a way that they are walking backwards. Mice were tested twice a day with a 45–60-min inter-trial interval for 5 consecutive days (same hour every day). For each day, the average time spent on the rotarod was calculated, or the time until the mouse made 3 consecutive wrappings/passive rotations on the rotarod (latency in seconds). Maximum duration of a trial was 5 min.

*Open Field test*. In this test, which is useful to test locomotor activity and anxiety, mice were individually placed in a brightly lit 110-cm diameter circular open field (25 lux in the middle of the arena) and allowed to explore the space for 10 min. The total distance moved by each mouse in the open arena was recorded by an infrared camera (Noldus® Wageningen, NL) connected to the EthoVision® software (Noldus® Wageningen, NL), and the outcome measure indicated as distance moved in centimeters.

*Marble burying test*. Open makrolon (polycarbonate) cages (50 × 26 × 18 cm) were provided with 4 cm of bedding material (Lignocel® Hygenic Animal Bedding, JRS). On top of the bedding material 20 blue glass marbles were placed in an equidistant 5 × 4 grid and the animal was placed in this cage for 30 min. The outcome measure is the number of buried marbles, which were scored as buried when covered by more than 50% by bedding material.

*Nest Building test.* Mice were single housed for a period of 5 to 7 days before the start of the experiment. Successively, the used nesting material was replaced with around 11 grams (11 ± 1) of compressed extra-thick blot filter paper (Bio-rad). The amount of the unused nesting material was weighed and noted daily for 5 consecutive days, each day at the same hour

*Forced swim test.* Mice were placed in a cylindrical transparent tank (27 cm high and 18 cm in diameter), filled with water (26 ± 1 °C) 15 cm deep for 6 min. The outcome measure is the time in seconds in which the mouse was immobile. The latency of immobility was only assessed during the last 4 min of the test. The mouse was considered to be immobile when it stopped moving, making only movements necessary to keep its head above water.

### Western blot analysis

To assess UBE3A expression, brain tissues were dissected and immediately stored in liquid nitrogen. The lysates were made by homogenization in lysis buffer (10 mM Tris-HCl, pH 6.8, 2.5% SDS, 2 mM EDTA) and supplemented with protease and phosphatase inhibitor cocktails (Sigma-Aldrich). Twenty micrograms of protein lysate was loaded on 4–12% SDS-PAGE gel (Bio-Rad) and transferred on nitrocellulose membranes to be then incubated with anti-UBE3A antibody (E8655 Sigma-Aldrich; 1:1000) and anti-actin antibody (MAB1501R, Millipore; 1: 20,000). Membranes were blocked in 4% TBS milk solution for 1 h at room temperature and incubated at 4 °C overnight, rotating end over end, with the primary antibody dissolved in 2% TBS-T milk solution. The day after, membranes were washed 3 times for 10 min with TBS-T and incubated with the secondary antibody, a fluorophore-conjugated goat anti-mouse antibody (IR Dye 800CW, Westburg; 1:15,000), dissolved in 2% TBS-T milk solution for 1 h. At the end of the incubation, membranes were washed 3 times for 10 min with TBS and the resulting blots were analyzed and quantified using a LI-COR Odyssey Scanner and Odyssey 3.0 software.

### Immunofluorescent and immunohistochemical staining

Mice were sedated with 0.15 ml Nembutal (60 mg/kg), transcardially perfused, and the brains were post-fixed with 4% paraformaldehyde in sodium phosphate buffer (PB) for 2 h. After incubation in 10% sucrose (in 0.1 M Phosphate buffer) overnight, brains were embedded in a sucrose/gelatin mixture (10 and 12%, respectively). Brain sections were cut on a microtome (SM2000R; Leica Microsystems, Rijswijk, Netherlands) at a thickness of 40 μm. The brain sections were then washed in PBS and incubated for 1 h in blocking buffer containing 10% normal horse serum (NHS) and 0.5% Triton X-100 in PBS. Subsequently, sections were incubated for 48–72 h in 2% NHS, 0.5% Triton X-100 diluted in PBS with primary antibody (mouse anti-E6AP, clone 3E5 Sigma-Aldrich; 1:750) and kept at 4 °C. For fluorescent stainings, sections were washed with PBS and the secondary antibody was added (anti-mouse Alexa 488, Jackson ImmunoResearch Labs, 1:200 diluted) in PBS containing 2% NHS and 0.5% Triton X-100. After 1–2 h incubation of the secondary antibody at room temperature, sections were washed in PB (0.05 M), mounted on, and covered using Mowiol (Sigma-Aldrich). Fluorescent images were acquired using the LSM700 confocal microscope (Zeiss). For DAB stainings, the secondary antibody (anti-mouse HRP, P0447 Dako; 1:200) was detected by 3,3-diaminobenzidine (DAB) as the chromogen, and DAB sections were imaged using a Nanozoomer scanner.

### Statistical analysis

All data were statistically analyzed using IBM SPSS software, and *P* values < 0.05 were considered significant. Statistical analysis was performed using univariate ANOVA or 2 way-repeated measures ANOVA with Bonferroni’s post hoc comparison. A Greenhouse-Geisser correction was used in repeated ANOVAs when the assumption of sphericity was not met.

For the Nestin-Cre experiments, a highly significant effect of genotype and highly significant interaction between presence and absence of Cre versus presence of absence of *Ube3a*^*mLSL*^ was observed for the nest building task, the marble burying test, and forced swim test (all *p* < 0.005). In line with previous power calculations, we did not have sufficient power to detect genotype differences for rotarod and open field when using four genotype groups [14]. To increase statistical power, we combined the WT Cre^+^ and Cre^−^ groups for subsequent statistical analysis, including the Bonferroni tests, which yielded a significant effect of genotype for rotarod. For the open field task, no significant effect of genotype could be observed.

## Results

### Characterization of the Nestin-CRE conditional AS mouse model

**Fig. 1:**
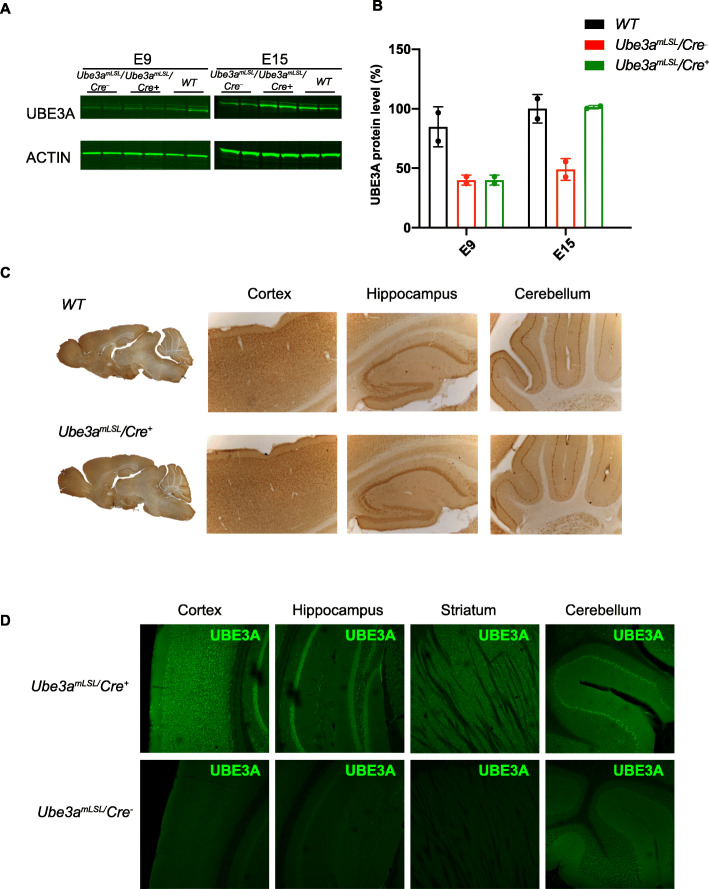
Successful maternal *Ube3a* gene activation upon Nestin-Cre-mediated deletion of the transcriptional STOP cassette. **a**, **b** Western blot data and UBE3A quantification from total lysates of E9 and E15 brains of *Ube3a*^*mLSL+*^, *Ube3a*^*mLSL/−*^ mice, and *WT* littermates normalized to WT E15 levels. Bars depict mean ± SD (*n* = 2 per genotype, per time point). **c**, **d** DAB and immunofluorescent pictures of UBE3A expression in sections of brains from adult wild-type, *Ube3a*^*mLSL*^*/Cre*^*+*^ and *Ube3a*^*mLSL*^*/Cre*^*−*^ *mice*

Previously we generated the inducible *Ube3a*^*LSL*^ line containing a floxed transcriptional “STOP-cassette” enabling UBE3A expression upon Cre-mediated deletion [10]. A partial rescue of behavioral phenotypes was observed when UBE3A expression was induced at postnatal day 1 (P1). However, since we achieved normal UBE3A gene expression in only 30% of the cells, a failure to rescue certain behaviors could also be attributed to a failure to induce gene expression in all cells. To further zoom in on this critical period for UBE3A functioning, we investigated if reactivation of *Ube3a* around the onset of the third week of mouse pregnancy would rescue AS phenotypes, thereby further narrowing down the critical window of UBE3A expression for full behavioral rescue. We took advantage of the Nestin-Cre line, in which Cre expression is under the control of the Nestin promoter [20], and crossed this line with the *Ube3a*^*LSL*^ line [10], resulting in *Ube3a*^*mLSL*^*/Cre*^*+*^ (Cre-positive) and *Ube3a*^*mLSL*^*/Cre*^*−*^ (Cre-negative) control mice. Nestin is an intermediate filament protein that is known as a neural stem/progenitor cell marker [23–25]. Since the Nestin promoter becomes active around E12.5 [26], we estimated that sufficient Cre expression to mediate deletion of the STOP cassette and to induce expression of UBE3A protein at wild-type levels would be reached around the start of the third week of mouse embryonic brain development. Indeed, Western blot analysis of brain UBE3A protein levels confirmed normal UBE3A protein levels in *Ube3a*^*mLSL*^*/Cre*^*+*^ mice at E15, whereas no expression of maternal UBE3A expression was observed in these mice at E9 (Fig. 1a, b). Consistent with the expression profile of Nestin, immunohistochemical and immunofluorescent stainings of adult *Ube3a*^*mLSL*^*/Cre*^*+*^ mice confirmed that UBE3A gene expression was obtained throughout the brain (Fig. 1c, d).

### UBE3A gene reactivation around the third week of mouse embryonic development prevents the manifestation of AS phenotypes

**Fig. 2:**
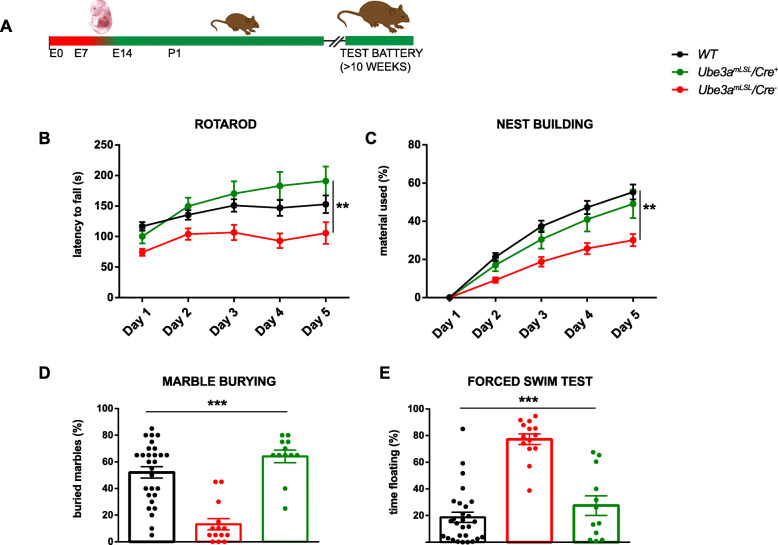
Behavioral testing of *Ube3a*^*mLSL*^/Nestin-Cre mice show a full rescue of most AS-like behavioral phenotypes. **a** Schematic representation of *Ube3a* gene reactivation during mouse embryonic development around 2 weeks of gestation and time point of behavioral testing. **b**–**e** Accelerating rotarod test, nest building test, marble burying test, and forced swim test in *WT* and *Ube3a*^*mLSL*^ mice (WT, *n* = 28; *Ube3a*^*mLSL*^*/Cre*^*−*^, *n* = 14; *Ube3a*^*mLSL*^*/Cre*^*+*^, *n* = 12). A repeated measures ANOVA or univariate ANOVA was used for statistical comparison of genotypes. Asterisk indicate significant effects of genotype: *P < 0.05; **P < 0.01; ***P < 0.001

After the biochemical validation of the new AS mouse line, we subjected wild-type mice (with and without Cre), *Ube3a*^*mLSL*^*/Cre*^*+*^*,* and *Ube3a*^*mLSL*^*/Cre*^*−*^ control mice to a well-characterized and robust behavioral testing battery [10, 14]. *Ube3a*^*mLSL*^*/Cre*^*−*^ control mice showed a significant deficit in all tasks except the open field task (*p* = 0.16) for which we previously showed that this is the weakest phenotype and for which we calculated that a sample size of 21 mice per group is required for sufficient power (1 − *β* = 0.95) [14]. In contrast, *Ube3a*^*mLSL*^*/Cre*^*+*^ mice, in which UBE3A expression was induced around the start of the last week of mouse embryonic development, were not significantly different from wild-type mice on rotarod (WT vs *Ube3a*^*mLSL*^*/Cre*^*+*^, p = 0.79), nest building (*p* value WT vs *Ube3a*^*mLSL*^*/Cre*^*+*^ = 0.8), marble burying (WT vs *Ube3a*^*mLSL*^*/Cre*^*+*^, p = 0.25), and the forced swim test (WT vs *U Ube3a*^*mLSL*^*/Cre*^*+*^, p = 0.65). This indicates that activating the maternal *Ube3a* allele around the onset of the last week of mouse embryonic development is sufficient to prevent the development of these phenotypes (Fig. 2b–e, Additional file 1: Table 1).

### Assessing paternal *Ube3a* expression during brain development

**Fig. 3:**
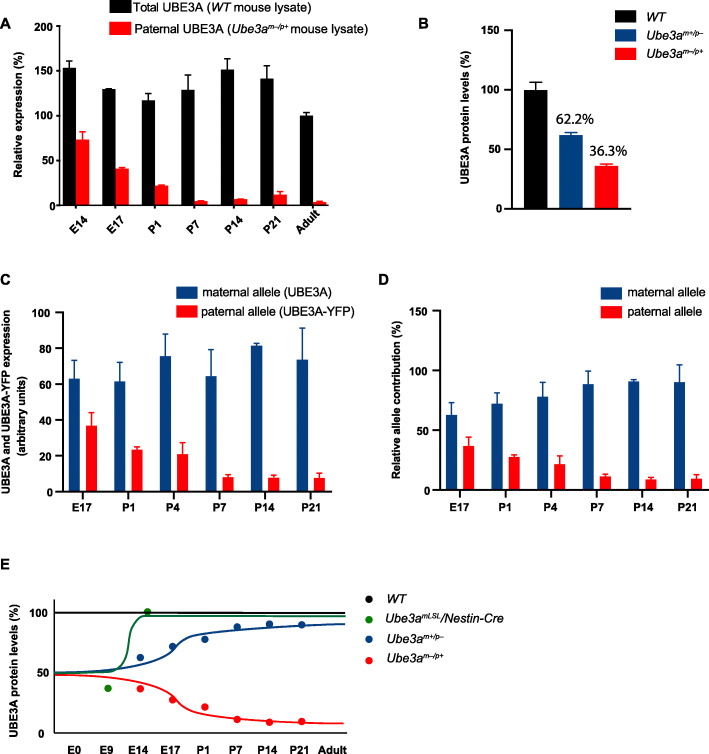
Silencing of the paternal *Ube3a* gene is not completed till after birth. **a** Total and paternal UBE3A expression during development. UBE3A was quantified upon Western blot analysis of total cortical lysates isolated from WT and AS (*Ube3a*^*m−/p+*^) mice at E14 (*n* = 8,5), E17 (*n* = 2,2), P1 (*n* = 6,3), P7 (*n* = 5,5), P14 (*n* = 4,3), P21 (*n* = 3,2), and adult mice (*n* = 2). Note that despite silencing of the paternal allele (red bars), total UBE3A levels remain rather constant (black bars), suggesting that the expression of the maternal allele increases at the same rate as the silencing of the paternal allele. All values are normalized against UBE3A levels in adult WT mice. Error bars represent mean ± SEM. **b** Loss of maternal UBE3A expression does not result in upregulation of paternal UBE3A expression. Note that the sum of UBE3A expression in *Ube3a*^*m−/p+*^ and *Ube3a*^*m+/p−*^ mice does not exceed 100%. UBE3A was quantified upon Western blot analysis of total lysates obtained from cortex isolated from WT (*n* = 5), *Ube3a*^*m−/p+*^ (*n* = 5), and *Ube3a*^*m+/p−*^ (*n* = 5) mice at E17. All values were normalized against UBE3A levels in WT mice. Error bars represent mean ± SEM. Absolute (**c**) and relative (**d**) expression levels of both alleles during development in *Ube3a*^*m+/pUBE3A-YFP*^ mice. **c** UBE3A and UBE3A-YFP signals were determined upon Western blot analysis of total lysates of cortex of *Ube3a*^*m+/pUBE3A-YFP*^ mice at E17 (*n* = 3), P1 (*n* = 2), P4 (*n* = 3), P7 (*n* = 3), P14 (*n* = 3), and P21 (*n* = 2). To enable direct comparison of both alleles, UBE3A-YFP immunoreactivity values of E17 *Ube3a*^*m+/pUBE3A-YFP*^ pups were calibrated against the UBE3A values of E17 *Ube3a*^*m−/p+*^ pups of **b**. The relative contribution of each allele was subsequently determined by calculating the contribution of each allele as percentage of both alleles for each time point (**d**). **e** Schematic representation of UBE3A levels in WT, *Ube3a*^*mLSL*^*/Nestin-Cre, Ube3a*^*m+/p−*^, and *Ube3a*^*m−/p+*^ mice, indicating that *Ube3a*^*m+/p−*^ mice can effectively be used as a UBE3A reinstatement model in which UBE3A levels are restored to normal levels after birth. The data points for this figure are based on the Western blots of **b** and **a**–**d**.

From the Western blot analysis of Fig. 1, it is clear that at E9, approximately 50% of UBE3A is still present in *Ube3a*^*mLSL*^*/Cre*^*−*^ mice. Given that the *Ube3a*^*mLSL*^*/Cre*^*−*^ mice and *Ube3a*^*mLSL*^*/Cre*^*+*^ mice show similar UBE3A levels, and that the Nestin promoter is not yet active at that time point, this protein must be derived from the paternal allele. Indeed, it has been shown that imprinting of the paternal allele is not completed till the first week after birth [11]. To investigate specifically the contribution of the paternal *Ube3a* allele during early brain development, we performed a Western blot on cortical lysates isolated from WT and AS (*Ube3a*^*m−/p+*^) mice at E14, E17, P1, P7, P14, P21, and adult mice. These results showed that UBE3A is entirely bi-allelically expressed at E14 (Fig. 3a), and only after birth the paternal allele gets fully silenced. Notably, UBE3A levels in wild-type mice remained relatively constant throughout development, despite increased silencing of the paternal allele. These results confirm previous findings that genomic imprinting does not serve to reduce the total amount of UBE3A [27].

Given that the total amount of UBE3A expression is not affected upon paternal silencing, expression of the maternal *Ube3a* allele must be increased during development to compensate for the loss of paternal expression. Likewise, there may be compensatory upregulation of the paternal allele in the absence of maternal *Ube3a* expression. Hence, it is possible that the level of paternal UBE3A expression as observed in AS (*Ube3a*^*m−/p+*^) embryos and pups (Fig. 3a) is increased as well. If such a compensatory mechanism is in place, we would expect that the total sum of UBE3A, as observed in *Ube3a*^*m−/p+*^ plus *Ube3a*^*m+/p−*^ mice, exceeds the amount of UBE3A as observed in wild-type mice. To test this, we crossed male *Ube3a*^*m+/p−*^ mice (*Ube3a*^*E113X*^ mutants) with female wild-type mice, as well as female *Ube3a*^*m+/p−*^ (*Ube3a*^*E113X*^ mutants) with male wild-type mice and quantified the amount of UBE3A protein in the mutant progeny at E17. As shown in Fig. 3b, the total sum of UBE3A protein produced in mice, in which the maternal or paternal *Ube3a* gene is deleted (99%), is comparable to the amount of UBE3A observed in wild-type mice. Hence, loss of the maternal *Ube3a* allele, does not result in compensatory upregulation of the paternal *Ube3a* allele.

Finally, we directly quantified the expression of both alleles within the same animals, by breeding mice in which the maternal allele expresses UBE3A and the paternal allele expresses UBE3A-YFP [22]. However, the values for UBE3A-YFP immunoreactivity are lower when compared to the values obtained when endogenous UBE3A is expressed from that same allele. Hence, we calibrated the paternal UBE3A-YFP values obtained from E17 pups, with the values obtained from E17 pups in which UBE3A was expressed exclusively from the paternal allele (as shown in Fig. 3b). The obtained results allow a direct comparison of both alleles during development, and confirm that full paternal *Ube3a* silencing is achieved in the first postnatal days of development (Fig. 3c, d).

### Contribution of paternal *Ube3a* expression to the behavioral phenotypes

We have previously shown for different *Ube3a* mutants that our behavioral testing battery is very sensitive to mutations affecting maternal *Ube3a* gene expression [10, 14, 28, 29]. Given the observation that the paternal allele is highly expressed throughout prenatal brain development (Fig. 3a–d), we wondered whether loss of paternal *Ube3a* expression (*Ube3a*^*m+/p−*^ mice) would also cause behavioral phenotypes on these tests. In fact, given the late onset of paternal silencing, *Ube3a*^*m+/p−*^ mice are conceptually comparable to a *Ube3a* gene reinstatement experiment in which we induce *Ube3a* gene activation around birth (see Fig. 3e for a graphical representation of this model).

Besides analyzing the effect of loss of paternal gene expression, we also wondered whether loss of both paternal and maternal *Ube3a* expression (*Ube3a*^*m−/p−*^) would worsen the behavioral phenotype compared to mice in which only the maternal *Ube3a* allele is deleted.

In order to disentangle the functional role of the maternal and paternal allele, we made use of two distinct *Ube3a* mutants that both cause loss of *Ube3a* expression: the commonly used *Ube3a*^*m−/p+*^ mice in which exon 5 is deleted (*Ube3a*^*tm1Alb*^; [12]) and the *Ube3a*^*E113X*^ mutant (*Ube3a*^*tm2Yelg*^; [21]) which carries a premature stop codon in exon 5. Both lines were shown to be similarly affected in all tasks of the behavioral testing battery [14]. Crossing these two lines with each other allowed us to determine whether the paternal or maternal allele was mutated in heterozygous offspring. This breeding yielded 4 experimental groups: WT mice, heterozygous *Ube3a*^*m+/*p−^ mice, *Ube3a*^*m−/p+*^ mice, and homozygous *Ube3a*^*m−/p−*^ mice.

**Fig. 4:**
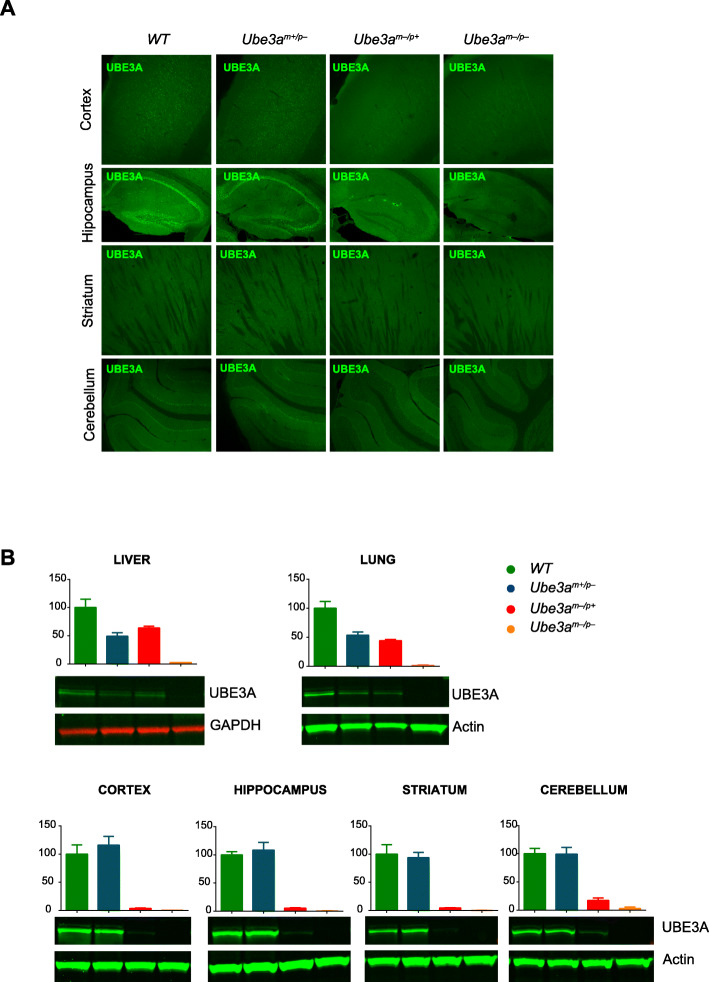
UBE3A expression in WT, *Ube3a*^*m+/p−*^ mice, *Ube3a*^*m−/p+*^ mice, and *Ube3a*^*m−/p−*^ mice. **a** Immunofluorescent staining of sections of cortex, hippocampus, striatum, and cerebellum of brains isolated from adult WT, *Ube3a*^*m+/p−*^ mice, *Ube3a*^*m−/p+*^ mice, and *Ube3a*^*m−/p−*^ mice. **b** Western blotting and UBE3A quantification in peripheral (liver and lung) and non-peripheral (cortex, hippocampus, striatum, and cerebellum) areas. Error bars represent mean ± SEM

Immunofluorescent staining of brains derived from adult mice of these 4 experimental groups shows that the expression of UBE3A in mice lacking the paternal allele (*Ube3a*^*m+/*p−^ mice) is comparable to wild-type mice (Fig. 4a). In contrast, adult mice lacking maternally derived UBE3A (*Ube3a*^*m−/p+*^ mice) show very low levels of UBE3A immunoreactivity, and even less staining was observed in the double mutants in which both maternal and paternal *Ube3a* alleles (*Ube3a*^*m−/p−*^ mice) are mutated (Fig. 4a). This was further confirmed by Western blot analysis of cortex, hippocampus, striatum, and cerebellum (Fig. 4b). In liver and lung, heterozygous *Ube3a*^*m+/*p−^ and *Ube3a*^*m−/p+*^ mice show comparable UBE3A expression, approximately 50% compared to wild-type levels. This confirms bi-allelic *Ube3a* expression outside the brain and, moreover, that both *Ube3a* mutant lines similarly affect *Ube3a* gene expression.

**Fig. 5:**
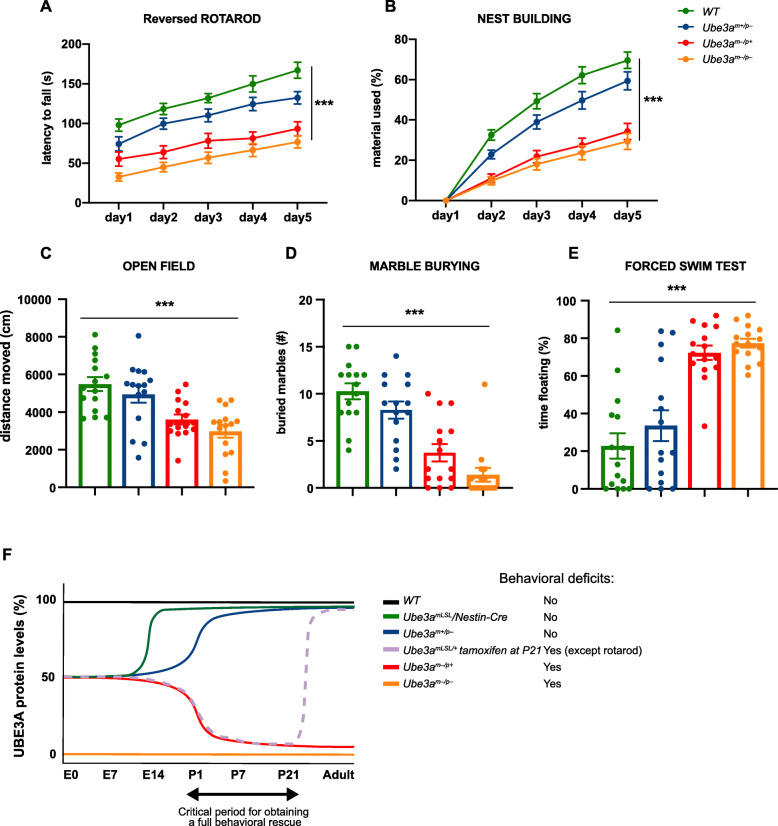
Limited function of the *Ube3a* paternal allele in the development of AS-like behavioral phenotypes. **a**–**e** Accelerating rotarod, nest building, open field, marble burying, and forced swim test in WT, *Ube3a*^*m+/p−*^, *Ube3a*^*m−/p+*^*,* and *Ube3a*^*m−/p−*^ mice (*n* = 15 per genotype). A repeated measures ANOVA or univariate ANOVA was used for statistical comparison of genotypes. Asterisks indicate significant effects of genotype, *p* < 0.001. Error bars represent mean ± SEM. **f** Schematic representation of several *Ube3a* lines used for studying the critical period, which indicates that the critical window of rescuing behavioral deficits in AS mice by gene reinstatement lies around birth and P21. Each curve depicts the level of UBE3A protein in WT, *Ube3a*^*mLSL*^*/Nestin-Cre, Ube3a*^*m+/p−*^, *Ube3a*^*m−/p+*^*,* and *Ube3a*^*m−/p−*^ mice (this study) over time. In addition, we included the previously published result of *Ube3a*^*mLSL*^ mice crossed with the inducible *Cag-Cre*^*ERT*^ line in which gene reactivation was induced by tamoxifen injection at P21 [10]. The presence of behavioral phenotypes has been indicated for each mouse line

The four experimental groups were then subjected to the behavioral test battery as described above. However, given the effect of paternal gene deletion on cerebellar maturation [12], we used the more difficult reversal rotarod task rather than the standard rotarod testing to increase the sensitivity of this task. *Ube3a*^*m−/p+*^ mice lacking the maternal allele showed a significant phenotype on all tests compared to wild-type littermate control mice. (Fig. 5, Additional file 1: Table 1). *Ube3a*^*m+/*p−^ mice lacking an active paternal *Ube3a* gene showed a tendency to display reduced performance compared to wild-type mice on all tests, but this never reached statistical significance. Similarly, *Ube3a*^*m−/p−*^ mutants in which both the maternal and paternal *Ube3a* allele were deleted, showed a tendency to impaired performance compared to *Ube3a*^*m−/p+*^ mice lacking only the maternal allele, but again this never reached statistical significance. This suggests that lack of paternal gene expression throughout prenatal brain development does not have a measurable effect on the phenotypes tested.

## Discussion

The purpose of this study was to further delineate the requirement of UBE3A expression during prenatal brain development. In order to address this question, we took advantage of a conditional mouse model of AS, in which the maternal *Ube3a* allele is reactivated around the onset of the third week of mouse embryonic development. In addition, we investigated the importance of expressing the paternal *Ube3a* allele during prenatal brain development, especially in terms of its contribution to the AS established phenotypes.

By reactivating *Ube3a* around the onset of the last week of mouse embryonic development, we were able to rescue all behavioral phenotypes in which AS mice were affected. These findings further narrow down the previously identified critical window for therapeutic intervention [10]. In our previous study [10], we identified a critical window for *Ube3a* reinstatement that lies between E0-P21. In this study, early embryonic reinstatement of *Ube3a* (E0) prevented AS phenotypes across different behavioral domains from developing. In contrast, postnatal reinstatement (P0–P21) rescued only few of the previously reported test battery phenotypes [10]. When we take our previous findings into account as well [10], our newest results suggest that the critical window for complete reversal of behavioral phenotypes can be narrowed down from the period around birth to P21 (Fig. 5f). It is notable that this period coincides with the period that UBE3A activity is controlled by PKA-mediated phosphorylation in mice and that loss of this phosphorylation due to a *UBE3A-p.T485A* mutation results in ASD in human [30].

We showed that during mouse embryonic development, UBE3A is expressed from both alleles. We found that around E14, expression is still bi-allelic, and full silencing of the paternal allele has not been achieved until the first week after birth, which is in line with previous findings [11]. We also observed that the total amount of UBE3A expression during development is not affected by paternal silencing, suggesting that there is a mechanism that increases maternal UBE3A expression at a comparable rate as the loss of paternal UBE3A expression. This observation is consistent with previous findings [27]. In contrast, we observed that loss of maternal UBE3A expression does not result in any compensatory expression of the paternal allele.

To further investigate the requirements of embryonic UBE3A expression levels, we made use of *Ube3a*^*m+/p−*^ mice in which the paternal *Ube3a* allele was mutated. In these mice, UBE3A protein levels were reduced to 50% during the first 2 weeks of embryonic brain development, and increased up to 80% of WT levels around birth. Analysis of these mice showed a tendency of decreased performance on all tests, but the effects were small and did not reach statistical significance. Hence, 50% of UBE3A protein is sufficient for normal prenatal brain development. Moreover, these experiments suggest that the critical period for UBE3A-dependent brain development in mice is restricted to the first few weeks after birth (Fig. 5f).

### Limitations

Our study has two major limitations. The first limitation is that we do not know how well the requirements for UBE3A expression during mouse brain development align with human brain development. The AS murine model has been widely used in the biomedical AS field to investigate potential therapeutic approaches, but despite the similarities between mouse and human, mice obviously have a much shorter lifespan compared to humans and timing of brain development and systems level functioning is very different. Several studies tried to investigate how we can correlate the two species in terms of brain development and function [31, 32]. When we align major developmental milestones, such as time to the weaning period or the age to attain puberty, 1 human year equals approximately 4 mouse days, and 57 mouse days, respectively [32]. A more elaborate comparison that includes brain anatomical milestones as well as major behavioral milestones indicates that mouse P1 compares to early second trimester in human [31, 32]. But if we aim to draw a parallel in identifying the best period for a behavioral rescue, it is particularly important to look at the critical periods underlying the development of sensory pathways, language, and higher cognitive functions [33, 34]. The best-understood critical periods controlling specific attributes of primary sensory modalities in animals are the representation of different tones in the auditory cortex or left versus right eye inputs in the visual cortex [34]. For this last case, the critical period for acquiring binocular vision closes around 4 weeks of age in mice, while in humans it remains open till 7 years of age [35]. Altogether, these studies indicate that it remains highly uncertain how we can precisely correlate the newly identified critical window to the human condition. But regardless of these limitations, our study suggests that early postnatal intervention is likely to be most successful to rescue AS related phenotypes and that the UBE3A gene plays a critical role in perinatal mouse brain development, but less so in adult mice [28].

The second limitation is that most of the behavioral tests we used have limited clinical value. With the exception of the rotarod, we do not know what brain areas are underlying the deficits in the open field test, marble burying test, nest building test, and forced swim test. These measures were selected because we have previously shown that they give robust phenotypes in different lines of AS mice and different genetic backgrounds [10, 14, 28, 29]. In addition we have previously calculated the power of these tests [14]. From that study, we know that we were underpowered to see a significant effect in the open field, which was indeed the case for the first set of experiments using the *Ube3a*^*mLSL/p+*^*/CRE*^*+*^ mice.

A minor limitation of our study is that although we did not find a statistically significant effect of paternal *Ube3a* gene deletion, all tests showed a tendency in the same direction. Hence, we cannot exclude a small effect, meaning that for optimal embryonic mouse brain development expression of both alleles is required. But nevertheless, our data implies that by losing 50% of protein expression before birth, the effect on the mouse behavioral phenotypes that we assessed is at best small.

## Conclusion

Our findings provide further important information about the requirements of *Ube3a* expression during brain development. Taken together with our previous studies [10, 28], our findings have further defined the critical period for obtaining a full behavioral rescue by *Ube3a* gene reinstatement strategies, and show that this period lies just after birth and before P21 [10] in AS mice (Fig. 5f). In addition, we show that loss of up to 50% of UBE3A protein during embryonic mouse brain development does not significantly impact the assessed mouse behavioral phenotypes.

## Supplementary information

**Additional file 1: Table 1.** Summary of the statistical tests used for the behavioral paradigms performed on each experimental group. Green cells indicate where a Greenhouse-Geisser correction was used in repeated ANOVAs when the assumption of sphericity was not met.

## Data Availability

The datasets used and/or analyzed during the current study are available from the corresponding author on reasonable request.

## Abbreviations

AS: Angelman syndrome
UBE3A: Ubiquitin protein ligase E3A
WT: Wild-type
PWS: Prader-Willi syndrome
AON/ASO: Antisense-oligonucleotide
